# Cancer Vaccines: Promising Therapeutics or an Unattainable Dream

**DOI:** 10.3390/vaccines9060668

**Published:** 2021-06-18

**Authors:** Howard Donninger, Chi Li, John W. Eaton, Kavitha Yaddanapudi

**Affiliations:** 1 James Graham Brown Cancer Center, Experimental Therapeutics Group, University of Louisville, Louisville, KY 40202, USA; howard.donninger@louisville.edu (H.D.); chi.li@louisville.edu (C.L.); 2 Department of Medicine, University of Louisville, Louisville, KY 40202, USA; eatonredox@aol.com; 3 Department of Pharmacology & Toxicology, University of Louisville, Louisville, KY 40202, USA; 4 James Graham Brown Cancer Center, Immuno-Oncology Group, University of Louisville, Louisville, KY 40202, USA; 5 Department of Surgery, Division of Immunotherapy, University of Louisville, Louisville, KY 40202, USA; 6 Department of Microbiology/Immunology, University of Louisville, Louisville, KY 40202, USA

**Keywords:** cancer vaccines, preventative vaccines, therapeutic vaccines, immunotherapy, personalized vaccines

## Abstract

The advent of cancer immunotherapy has revolutionized the field of cancer treatment and offers cancer patients new hope. Although this therapy has proved highly successful for some patients, its efficacy is not all encompassing and several cancer types do not respond. Cancer vaccines offer an alternate approach to promote anti-tumor immunity that differ in their mode of action from antibody-based therapies. Cancer vaccines serve to balance the equilibrium of the crosstalk between the tumor cells and the host immune system. Recent advances in understanding the nature of tumor-mediated tolerogenicity and antigen presentation has aided in the identification of tumor antigens that have the potential to enhance anti-tumor immunity. Cancer vaccines can either be prophylactic (preventative) or therapeutic (curative). An exciting option for therapeutic vaccines is the emergence of personalized vaccines, which are tailor-made and specific for tumor type and individual patient. This review summarizes the current standing of the most promising vaccine strategies with respect to their development and clinical efficacy. We also discuss prospects for future development of stem cell-based prophylactic vaccines.

## 1. Introduction

The recent development of several effective vaccines against SARS-CoV-2 (COVID-19) was greeted with great relief, short though of balloons and parades. Given that repeat infections may occur despite immune-mediated clearance of the first infection, doubts remain regarding whether durable immunity will be achieved in all vaccinated people. COVID-19 vaccines in use and in development vary widely in mode of delivery and composition (multivalent vs. monovalent). In contrast, two of the oldest vaccines against small pox and polio are polyvalent and have proved effective in disease prevention. Despite the enormous success of prophylactic vaccination against pathogens, there are currently no vaccinations available for providing pre-emptive immunity against cancer.

The “antigenic drift” that occurs in tumor cells helps them to elude the antibody-mediated clearance limiting the efficacy of vaccines that are developed using a classical vaccine platform. In addition, high disease burden, immunosuppressive and regulatory mechanisms come into play and evolve during cancer progression in the tumor-bearing hosts as well as within the tumors, further undermining the efficacy of vaccines. In the majority of tumor-bearing hosts, the innate and adaptive immune response work in unison to mediate tumor cell destruction. However, specific types of immune cells (e.g., T regulatory cells [1], tumor-associated macrophages [2] and myeloid-derived suppressor cells [3]) actively dampen the host immune responses against the tumor; expansion of these cells promotes tumor-growth and non-responsiveness to cancer immunotherapies. In spite of these limitations, the field of therapeutic and prophylactic cancer vaccines is fast evolving, and considerable progress has been achieved with the clinical investigation of several of these anti-cancer vaccine agents. The approval of Provenge^®^ (sipuleucel-T), a dendritic cell-based vaccine for the treatment of prostate cancer a decade ago [4], was significantly encouraging for the field of therapeutic cancer vaccines. This success has highlighted the fact that anticancer vaccines can work and has provided incentive for further investment in cancer vaccine development.

This review discusses various cancer vaccine strategies aimed at enhancing activation of anti-tumor immune responses (outlined in Table 1). We discuss the current status and the recent advances made in the field of vaccine-based immunotherapy of tumors, and report on progress in developing an alternative polyvalent vaccine which employs the well-known similarities between embryos and many cancers (the so-called carcinoembryonic antigens). Given the ubiquitous nature of such shared antigens, such a vaccine has the potential to protect against a variety of different neoplastic diseases.

## 2. Preventive and Therapeutic Cancer Vaccines

Scientific advances during the two centuries since the discovery of the smallpox vaccine by Edward Jenner have led to the development of several prophylactic, i.e., preventive vaccine programs against infectious diseases with numerous successes. These successes are attributed to the fact that the causative agents of most infectious diseases are known and are recognized by the immune system as “non-self”. The development of anti-tumor vaccines, however, is problematic, mainly because the tumors developing in host tissues largely express “self” antigens, to which the immune system has previously been tolerized. These tissue-specific self-proteins are frequently expressed in numerous normal tissues making them unsuitable targets for anti-cancer vaccine development. Recently, several non-tolerogenic, tumor-associated antigens (TAAs) have been identified and are attractive targets for the development of anti-cancer vaccines. Cancer cells are equipped with mechanisms that allow them to evade recognition by the immune system or to suppress the functionality of cancer-fighting T cells. Therapeutic cancer vaccines using TAAs aim to stimulate anti-tumor T cell responses or to block regulatory mechanisms that suppress the function of tumor-reactive T cells, thereby tipping the balance from a pro-tumoral to an anti-tumoral immune environment that, ultimately, will result in reduced tumor growth. Recent progress made in understanding the molecular mechanisms used by tumor cells to escape immune surveillance is aiding the development of therapeutic and preventive cancer vaccines that could be effective against a variety of human cancers that are not associated with any known etiopathogenic agent.

*Prophylactic Cancer Vaccines.* Prophylactic vaccines are designed to prevent a cancer from establishing itself and have proven to be successful in reducing the global burden associated with two cancer-causing viruses, Hepatitis B Virus (HBV) [5] and Human Papilloma Virus (HPV) [6,7]. The US Food and Drug Administration (US FDA) has approved vaccines for cancer-associated with these viral infections.

The anti-HBV vaccine was the first anti-cancer vaccine to be implemented in the clinic after its approval in 1981. Persisting HBV infection, which is more frequent if the virus is acquired earlier during childhood, carries a substantial risk of progressive cirrhosis, liver failure and hepatocellular carcinoma. One-third of the million deaths due to chronic HBV infection can be attributed to hepatocellular carcinoma [8]. Today, the worldwide incidence of hepatitis-associated hepatocellular cancer is decreasing as most children in the United States and in other parts of the world are being vaccinated. The highly immunogenic anti-HBV vaccine containing the recombinant HBV surface antigen (HBsAg) has been shown to convey lifelong immunity [9]. The development of vaccines for other common viral infections associated with cancer, i.e., hepatitis C virus (HCV), Epstein-Barr virus (ECV) and Helicobacter pylori are hindered by the genomic instability and the incomplete understanding of protective immune responses.

Two anti-HPV vaccines have been approved by the US FDA. Multiple pathological strains of HPV are responsible for around 70% of all cervical cancers worldwide and some cancers of the vulva, vagina, oropharynx and anus. Gardasil^®^, manufactured by Merck, and Cervarix^®^, manufactured by GlaxoSmithKline, confer protection against HPV (types 16 and 18) [10]. Both vaccines are derived from viral subunit-like particles (VLPs) composed of the single capsid protein L1. The efficacies of these two vaccines rely on the generation of a strong neutralizing antibody response against immunomodulant viral antigens. The bivalent Cervarix^®^ is composed of proteins from HPV-16 and HPV-18, while the quadrivalent Gardasil^®^ contains VLPs from HPV-6, HPV-11, HPV-16, and HPV-18 [11]. Recently, the FDA approved the use of a 9-valent HPV vaccine (Gardasil 9) for use in adults aged 27–45 years. Both vaccines present an excellent safety profile, are highly immunogenic, and confer complete protection against persistent infection in vaccinated women. Some unresolved issues remain; these include identification of the most critical groups of individuals and improving the efficiencies of vaccine production so that they can be deployed in developing countries.

The successful deployment of HBV and HPV vaccination programs for the prevention of viral-associated cancer has demonstrated that effective prophylactic vaccines can reduce the global burden of cancer. As it stands, exploring new vaccine strategies that can induce anti-tumoral cellular immune responses as well as neutralizing antibodies appears to be required for the achievement of robust protection against these cancer-inducing agents.

Despite the enormous success of prophylactic vaccination against pathogens as well as against virus-associated cancers, there is currently no vaccine for providing prophylactic immunity against adult-onset non-viral-associated cancers, such as breast cancer and ovarian cancer. Recent studies by Tuohy’s group have proposed that anti-cancer immunity in cancer-free subjects can be generated by immunotherapeutic targeting of certain tissue-specific self-proteins that are “retired antigens”, i.e., immunodominant antigens that are no longer expressed in normal tissues as a result of host’s normal aging process but are expressed by tumor cells that emerge due to aging [12]. Examples of such cancers include breast, ovarian and prostate cancers; retired antigens are attractive targets for preventing the occurrence of these cancers in otherwise healthy subjects (primary immunoprevention). The authors have identified two such retired antigens, extracellular domain of anti-Mullerian hormone receptor II (AMHR2-ED) [13] and α-lactalbumin proteins [14]. Both of these antigens are expressed in cancer cells but show limited expression in normal tissues. α-Lactalbumin protein is normally expressed only in human lactating breast tissue but is overexpressed in triple-negative breast cancer [15]. Similarly, human AMHR2-ED gene expression occurs in the ovary and adrenal gland and its expression declines over age; ovarian AMHR2-ED gene expression is significantly lower in postmenopausal ovaries compared to premenopausal ovaries [12]. In a preclinical study using mouse models of ovarian cancer, prophylactic vaccination of mice against AMHR2-ED antigen induced effective inhibition in the growth of both autochthonous and transplantable epithelial ovarian carcinoma (EOC) [13]. In this study, AMHR2-ED inhibited the growth of EOC by activating CD4+ T helper cells that in turn facilitated B cells to produce AMHR2-ED-specific IgG that activated a Bax/caspase-3 dependent proapoptotic signaling cascade ultimately inhibiting the growth of EOC cells. Future studies should test the safety and efficacy of immunoprevention vaccine agents (with retired agents) in human clinical trials for immunoprevention of these diseases.

Primary immunoprevention of breast cancer, ovarian cancer, prostate cancer and other adult-onset cancers is a great unmet need in oncology; development and clinical testing of prophylactic agents that perhaps prevent oncogenesis will greatly help to reduce mortality due to these deadly diseases.

*Immunotherapeutic Vaccines.* Therapeutic cancer vaccines have been tested in the clinic for several decades. Several different categories of therapeutic cancer vaccines are currently being evaluated. These include cellular (whole tumor/immune cells), viral vector, or molecular (peptide, TAA-encoding DNA, or RNA). The therapeutic efficacy of these agents has been improved by using the optimal formulation for vaccine delivery and by co-administration of immunological adjuvants, immune stimulatory cytokines and addition of costimulatory molecules. Recent advances made in vaccine formulations have led to the development of multiple novel immunotherapy interventions that work to effectively promote antigen presentation, activate effector T cells and overcome tumor-induced immunosuppressive mechanisms. Unfortunately, many of these immunotherapeutic approaches have failed to generate significant clinical benefits in the therapeutic setting. In 2010, the FDA approved the first vaccine, sipuleucel-T (Provenge^®^, commercialized by Dendreon, Inc.), for use in some patients with metastatic, castration-resistant prostate cancer. Currently, the most advanced therapeutic paradigm is personalized cancer vaccines that are tailored to react to tumor-specific antigens expressed by an individual patient. The increased access to cutting-edge next-generation sequencing technologies and bioinformatics pipelines, offers enormous possibilities for developing these cancer patient-specific immunotherapeutic vaccines that show great promise in clinical settings.

*(A) Immunotherapeutic Whole-cell and Peptide Vaccines*. The majority of cancer immunotherapies developed are directed towards activating the adaptive immune arm, particularly the cytotoxic CD8^+^ T effector cell compartment, of the immune system. Success of a cancer vaccine relies on the antigen processing capabilities of the APCs and their effective transport to draining nodes, a site wherein the presented TAAs activate tumor-reactive cytotoxic CD8^+^ T cells. The goal of any cancer therapeutic vaccine is to increase the pool of tumor-specific T cells from the population of naïve T cells, as well as to reactivate existing tumor-specific T cells that may be in an anergic state. In order to achieve these goals, defined tumor-specific antigens (TAAs) are required to stimulate dendritic cells (DCs) to optimally activate T cells. A major drawback is that the CD4^+^/CD8^+^ T cells against many of these antigens have been removed from the immune cell repertoire by central or peripheral tolerance [16,17,18,19,20]. Therefore, the selection of TAAs as vaccine targets requires careful consideration and any cancer vaccine should possess the ability to stimulate or reactivate low affinity or rare TAA-reactive T cells [18,21].

*Whole-cell vaccines.* Perhaps the most rational way of achieving this goal, while simultaneously overcoming the major obstacle of immune tolerance and identification of rare TAAs, is the use of whole tumor-cell preparations, which would target the broadest range of TAAs. Strategies in this approach include the use of autologous tumor lysates, irradiated autologous tumor cells or allogeneic tumor cell lines.

Early vaccines comprised of irradiated whole tumor cells alone proved not to be very effective. However, the use of several cytokines, such as interleukin-2 (IL-2), interferon α (IFNα) and granulocyte-macrophage colony-stimulating factor (GM-CSF) as adjuvants led to the development of genetically modified whole tumor cell strategies which has increased the efficacy of whole cell vaccines. Perhaps the best studied of these are the GVAX vaccines, which consist of either autologous or allogeneic tumor cells genetically modified to overexpress GM-CSF [22,23]. GM-CSF attracts antigen presenting cells (APCs), mainly dendritic cells, to the site of vaccination where they internalize antigens released from the apoptotic tumor cells. These dendritic cells then migrate to the draining lymph nodes where they present the TAAs to effector T cells and activate them [24]. While these vaccines show promise in preclinical models where they induce potent immune responses and tumor regression [25,26,27,28], their efficacy in clinical trials for a variety of human tumors is unexpectedly low even though they stimulate immune responses. In a phase 1/2 clinical trial of hormone refractory prostate cancer, GVAX derived from prostate cancer cells lines (Cell Genesys, Inc. [29]) showed only a modest delay in disease progression [30]. Using a different vaccine derived from 3 allogeneic prostate cancer cell lines which were selected to represent tumor found at the major sites of the disease [31], an additional clinical trial found more efficacious results with a median time to disease progression of 58 weeks [31]. Unfortunately, two subsequent phase 3 trials using GVAX in chemotherapy-naïve prostate cancer patients were terminated early and this therapeutic approach has not been developed further for prostate cancer [32]. However, a cancer vaccine is available for prostate cancer patients. Sipuleucel-T (Provenge^®^) is the first FDA-approved cancer vaccine and is currently in use for metastatic castration resistant prostate cancer [4]. This vaccine is an autologous dendritic cell-based vaccine loaded with a prostatic acid phosphatase (PAP) antigen fused to GM-CSF. Although the precise mechanism of action of Provenge^®^ is unknown, it is likely that APCs taking up the PAP-GM-CSF fusion protein will stimulate CD8+ T-cells to target PAP-expressing prostate tumor cells specifically [4]. A pivotal phase 3 study (IMPACT) showed that Provenge treatment provided a small but significant increase in overall survival as compared to placebo [33] however, the cost of Provenge and the complexity of its production are hurdles for its widespread use [22]. Nonetheless, Provenge demonstrates that it is possible to successfully create an autologous DC-based vaccine that works. While this vaccine has shown success and has been FDA-approved, it is currently rarely used due to complex vaccine production process resulting in high cost of production and issues with reimbursement by health insurance companies. Moreover, EMA approval for Provenge was withdrawn in 2015 [34] resulting in limited global availability.

Whole cell vaccines are also currently being extensively evaluated in pancreatic cancer patients with a number of ongoing and completed clinical trials [35,36]. GVAX, comprising two human allogeneic pancreatic tumor cell lines [37], is again the most widely studied. In early clinical trials, GVAX alone or in combination with other chemotherapeutic regimens, exhibited increased disease-free survival and better clinical outcomes [37,38,39], however, in other clinical trials for pancreatic cancer, results were disappointing [36]. A further whole cell vaccine that has been tested in pancreatic cancer is Algenpantucel-L (NewLink Genetics Corporation) which is composed of 2 irradiated human pancreatic ductal adenocarcinoma cell lines (HAPa-1 and HAPa-2) that have been genetically engineered to express the murine enzyme α(1,3)-galactosyltransferase [αGT, [40]]. The αGal epitope is not expressed on human cells due to inactivation of the *αGT* gene [41], however, human lymphocytes produce large quantities of the anti-αGal antibody which results in hyperacute rejection, a form of rejection occurring rapidly after non-human allograft transplantation [42,43]. Algenpantucel-L elicits an immune response by first triggering hyperacute rejection and phagocytosis of the αGal epitopes on the vaccine tumor cells, followed by the education of the patient’s immune effector cells to recognize other TAAs expressed by the vaccinated cells, a phenomenon first identified in preclinical mouse models [44,45]. Unfortunately, in a phase 3 Impress clinical trial, Algenpantucel-L provided no additional benefit and showed no improvement in overall survival for patients with resected pancreatic cancer, and no subsequent trials are planned due to the lack of efficacy of this therapeutic vaccine [35].

Cellular vaccines have also been extensively studied for melanoma. Clinical trials employing GM-CSF expressing allogeneic tumor cells for melanoma have largely been conducted on late stage cancer patients [46], and again, despite inducing potent antitumor immunity, this strategy offers minimal advantages in terms of clinical outcome [47,48,49,50]. In a slightly unconventional approach, a phase 1 clinical trial to test the efficacy of whole tumor cell vaccine in patients with non-small cell lung cancer (NSCLC) used autologous GM-CSF-secreting whole cell vaccines generated from resected metastases from the individual patients [51]. These vaccines again elicited strong anti-tumoral immune responses but had limited efficacy in terms of patient outcome [51]. In addition to this approach, other cellular vaccines have been developed for NSCLC, including belagenpumatucel-L (Lucanix™), an allogeneic tumor cell vaccine, and GVAX, an autologous tumor cell vaccine [52]. Although in phase 2 trials belagenpumatucel-L correlated with an increased survival when administered at high doses [53,54], there was no difference in survival between patients receiving belagenpumatucel-L or placebo in a phase 3 trial investigating its use as a maintenance therapy in late stage NSCLC patients, and the trial was prematurely stopped [55].

A novel strategy for cell-based vaccines uses cyclic dinucleotides (CDNs) formulated with irradiated GM-CSF-secreting whole cell vaccines, termed STINGVAX [56]. CDNs were initially classified as bacterial second messengers that activate the TBK1/interferon regulatory factor 3 (IRF3)/type 1 interferon (IFN) signaling axis via the cytoplasmic pattern recognition receptor stimulator of interferon genes (STING) [57]. Activation of the STING pathway elicits secretion of type I interferons and proinflammatory cytokines and has the potential to enhance the efficacy of immunotherapy [57]. In preclinical studies, STINGVAX activated dendritic cells (DCs), enhanced tumor-infiltrating lymphocytes (TILS) and in combination with PD-1 blockade, induced tumor regression of established tumors [56]. This strategy, however, has not been tested in cancer patients as yet, thus its true clinical benefits remain to be determined.

In addition to T-cell priming, whole cell vaccines also induce antibody-mediated immune responses [58]. Mice immunized with a whole cell vaccine against 4T1 mammary tumor cells exhibited higher titers of serum IgG than non-vaccinated mice, and these titers correlated with T-cell responses [58]. However, whole-cell vaccines induce large IgG and IgM responses to non-human antigens that are components of the systems used to produce the vaccines, such as fetal bovine serum (FBS), and these responses to the non-human antigens outcompeted the productive response to the vaccine and correlate inversely to patient survival [59]. Thus, careful assessment of vaccine production components is critical to maximize the efficacy of whole-cell vaccines.

*Peptide-based vaccines*. An alternative approach to whole cell vaccines are peptide-based vaccines. This strategy is based on the fact that T cells recognize cancer associated antigens in a similar manner to how they recognize virally infected cells that is they recognize intracellular antigenic peptides presented by MHC molecules on the surface of antigen presenting cells (APCs). Scientific advances in the 1980’s in the field of cloning technology along with studies involving the screening of patient tumor–derived expression libraries with autologous tumor-reactive T cells helped to define the molecular identity of TAAs that are recognized by spontaneous T cell responses [60,61]. Two different types of TAAs have been identified (i) non-mutated TAAs and (ii) proteins that are translated from mutated genes. Non-mutated TAAs include antigens upregulated during malignant transformation (e.g., oncofetal antigens, carcinoembryonic antigen, α-fetoprotein), developmental antigens (e.g., MAGE, tyrosinase, melan-A, gp100); cancer/testis antigen (NY-ESO-1), and viral antigens associated with oncogenesis. Certain solid tumors (e.g., melanoma, lung cancer) are characterized by an accumulation of genetic alterations that are caused by exposure to mutagens such as ultraviolet light [62] and carcinogens in cigarette smoke [63]. The advantage of targeting TAAs is that they are frequently expressed in the tumors of multiple individuals, however, the disadvantage is that TAAs often display limited antigenicity due to the fact that they are self-antigens, resulting in a lower repertoire of high affinity T cells specific for these antigens due to immune tolerance [20,64].

The most important aspect of peptide-based cancer vaccines is the choice of tumor antigen. A multitude of TAAs have been identified and used in the development of therapeutic cancer vaccines, including, but not limited to cancer/testis antigen 1B (CTAG1B, better known as NY-ESO-1 [65,66,67]), MAGE family member A3 (MAGE-A3, [68,69,70,71]), baculoviral IAP repeat containing 5 (BIRC5, otherwise known as survivin, [72,73,74]) and Wilms’ Tumor 1 (WT1, [75,76]). For a more complete list of TAAs under development and in use as peptide vaccines see Bezu et al. [77].

Peptide-based vaccines have been tested in a myriad of clinical trials for a variety of cancers (for a comprehensive list of clinical studies see Bezu et al. [77]) and although they are generally well tolerated and can elicit anti-tumor immune responses, peptide vaccines used as standalone interventions remain largely ineffective [77,78] and several clinical trials have been prematurely terminated for various reasons. For example, a MAGE-A3 immunotherapeutic comprising recombinant MAGE-A3 protein given with a proprietary adjuvant, A15 (GlaxoSmithKline), showed promising clinical benefit in a phase 2 study in melanoma patients [79]. These results, together with results from a further phase 2 clinical trial of the MAGE-A3 immunotherapeutic in NSCLC patients [80], were deemed sufficient to initiate a global, multicenter, phase 3 study (DERMA) in melanoma patients [71]. However, data from this study showed no clinical benefit of the vaccine and based on these negative results, development of MAGE-A3 as an immunotherapeutic for melanoma has been stopped [71]. A further peptide-based vaccine, Tecemotide, a MUC-1 targeting vaccine [81], also initially showed promise in a phase 3 trial (START) for unresectable stage III NSCLC treated concurrently with chemoradiotherapy [82], however, 2 additional clinical trials (START2; NCT02049151 and NCT01423760; www.clinicaltrials.gov, accessed on 20 May 2021) were halted as the sponsor discontinued Tecemotide in NSCLC. A Phase 2 trial, testing a multi-epitope peptide-based vaccine in combination with daclizumab with or without recombinant IL-12 (NCT01307618, www.clinicaltrials.gov, accessed on 20 May 2021), was also terminated prematurely due to lack of efficacy. Consequently, no peptide-based vaccines are currently approved by the US Food and Drug Administration (FDA) or equivalent worldwide agencies [77].

Perhaps peptide-based vaccines will garner more success when used in combination with other immunological adjuvants and therapeutic strategies. Currently, there are a number of ongoing clinical trials testing the efficacy of peptide-based vaccines in combinations with several adjuvants such as Montanide ISA-51 [83,84], Hiltonol^®^ (poly-L-lysine in carboxymethylcellulose, a TLR3 ligand, [85]) as well as in conjunction with other therapeutic regimens, such as conventional chemotherapy, radiation, targeted cancer therapeutics and other immunotherapeutic approaches (immune checkpoint inhibitors and immunostimulatory antibodies [77]) and other immunomodulatory agents such as lenalidomide [86,87]. Thus, while peptide vaccines are safe, well tolerated and can generate anti-tumor immune responses, it is clear that much work still remains in order to develop a clinically beneficial peptide-based vaccine whose efficacy rivals that of current immunotherapeutic options.

*(B) Personalized Cancer Vaccines.* Ernest Tyzzer, in 1916, introduced the term “somatic mutations,” and described how cancer cells use these mutations to acquire new immunogenic properties [88]. The Cancer Genomic Projects and the ensuing research have led to the discovery of genetic alterations called driver gene mutations, which include both gene mutations and gene rearrangements; these somatic mutations promote oncogenesis [89]. The second kind of somatic mutations are called the passenger mutations and these mutations are generally not attributed to any functional relevance [89]. However, both these somatic mutations can alter the sequence of proteins and these sequence-altered proteins are called “neoantigens”. The cancer-specific neoantigens are processed and presented on major histocompatibility complex (MHC) molecules and these mutated epitopes that are recognized by T cells are called “neoepitopes” [90]. Neoepitopes are not expressed on the normal tissue and hence are recognized as foreign by the host’s immune system, and thus can elicit T cell immunity against tumors [60,90,91].

Previously published data in melanoma patients treated with immune checkpoint blockade therapies and adoptive transfer of autologous tumor-infiltrating lymphocytes (TILs) indicate that higher nonsynonymous tumor mutation burden and neoepitope-specific T cells strongly associate with durable clinical benefit [92,93,94]. Not only do mutation rates generally predict immunotherapy responsive cancer types (i.e., T cell enriched “hot” tumors like melanoma and non-small cell lung carcinoma [95,96], but they can also predict response/resistance associations within individual immunotherapy-treated patients [97,98]. Next-Generation Sequencing (NGS) of the entire tumor “mutanome”, i.e., mapping of all the mutations in the tumors and prediction of MHC molecule–binding neoepitopes revealed that tumor mutation burden and T cell infiltrations correlate with patient survival across various cancer types [95,99]. These findings are consistent with the hypothesis that a high mutational load and the presence of a higher diversity of T cells that recognize the neoepitopes prior to therapy is a requirement for neoantigen-based immunotherapy. The paradigm of personalized cancer immunotherapeutic vaccine approach is designed to utilize these foreign neoepitopes generated from the somatic mutations as cancer-specific targets. One of the challenges of such a personalized vaccine strategy is the fact that every patient’s tumor displays a unique set of mutations and these need to be identified using next-generation sequencing (NGS) approaches. A number of recent preclinical and clinical studies have proposed such a personalized vaccination approach to identify and target the entire spectrum of tumor-specific mutations present in an individual tumor of a cancer-bearing hosts.

*Neoantigens-based personalized vaccine strategies*. Neoantigen-based personalized vaccines can be in various formats including mRNA-, peptide- and whole cell-based strategies.

*(i) mRNA vaccines.* In one such preclinical study, Kreiter et al. used three independent murine tumor models and show that the majority of the non-synonymous tumor mutations are recognized by CD4^+^ T cells and vaccination with such CD4^+^ immunogenic mutations induce potent anti-tumor responses [100]. The authors used NGS and bioinformatics and established a pipeline to select mutations as vaccine targets by prioritizing their expression levels and MHC class II-binding capabilities. Such a pipeline was used to produce synthetic poly-neo-epitope messenger RNA vaccines [100]. Vaccination of tumor-bearing mice with such polytope mRNA vaccines induced almost complete rejection of established tumors in vaccinated mice. Furthermore, this study showed that CD4^+^ T cell neo-epitope vaccination induced CD8^+^ CTL responses against an independent tumor antigen in the vaccinated mice, possibly due to the occurrence of an “antigenic spread” in the vaccinated mice [100].

*(ii) Peptide vaccines.* In a similar preclinical study, an NGS and MHC Class I prediction model was used to identify MHC Class I-restricted CD8^+^ T cell reactive neoepitopes in a mouse sarcoma model [101]. The neoepitope-specific CD8^+^ T cells were present in progressively growing sarcoma tumors and could be reactivated following treatment with immune checkpoint therapies [101]. Interestingly, vaccination with long synthetic peptides of the identified neoepitopes activated anti-tumoral CD8^+^ T cell immunity in the vaccinated mice [101]. Duan et al. identified two novel tools that can accurately predict neoepitopes that are capable of mounting an anti-tumor immune response in the cancer-bearing hosts [102]. The authors tested the anti-tumor activity of mutated 9-mer peptides in tumor-bearing mice. The tools calculated the difference in predicted affinity for a given mutant and wild-type peptide pair and the predicted stability of MHC class I/peptide. These scores were both used to determine the odds of a protective neoepitope (peptide) to be recognized by CD8^+^ T cells [102]. In another preclinical study, whole-exome and transcriptome sequencing in combination with mass spectrometry was used to identify MHC Class I-binding neoepitopes in two murine tumor models [103].

In a clinical study, six melanoma patients with resected tumors (stage III n = 4; stage IV n = 2 patients) were vaccinated with long peptides derived from 20 neoantigens/mutations per patient along with an adjuvant mixture that consisted of toll-like receptor 3 (TLR3) and melanoma differentiation-associated protein 5 (MDA-5) [104]. Data from this trial showed that all the four stage III patients remained cancer-free with no disease recurrence, while the two metastatic patients who did relapse were found to be very responsive to anti-programmed cell death protein 1 (anti-PD-1) therapy. In another clinical trial [105], thirteen stage III/IV melanoma patients were injected with a personalized RNA-based mutanome vaccine encoding ten of their individual mutations as long 27-mer peptides. The results from this trial were promising and showed that eight patients experienced prolonged disease-free survival and among the remaining five patients with metastatic disease at the time of vaccination, two patients experienced objective responses. With respect to the induction of immunological T cell responses, both of these vaccination trials involve long peptides and thus, rely on the peptide processing and presentation of neoepitopes by the patient’s APCs to determine which portion of the peptide is presented for T cell receptor recognition and induction of tumor-reactive T cell responses. Data from both these studies showed expansion of preexisting T cells, of which CD4^+^ T cells occurred at a higher proportion in response to the vaccination, and some peptides activated both CD4^+^ T and CD8^+^ T cell responses. These studies provide evidence that vaccines against tumor neoantigen-derived immunogenic peptides resulting from somatic mutations can be utilized to activate patient-specific tumor-reactive immune responses and thus, can be therapeutically effective.

*(iii) Whole cell vaccines.* Importantly, the concept of patient-specific mutanome vaccines was next tested in first-in-human studies in cancer patients. In one such study [106], three melanoma patients were vaccinated with autologous Dendritic cells (DCs) loaded ex vivo with synthetic 9-mer HLA class I-associated peptides derived from tumor-associated neoepitopes derived from individual mutations unique to each patient. The mutated peptides that are formulated into this vaccine were restricted by HLA-A*-02 molecules, the most frequently present class I haplotype. The results from this small phase I trial suggested that expansion of CD8^+^ T cells specific to mutant peptides did occur in all the patients. However, the tumor-killing functions of these tumor-reactive CD8^+^ T cells were not assessed and whether this personalized DC-based vaccine strategy is capable of improving patient cancer-free survival remains to be assessed in future clinical trials.

*Combination of non-mutated and mutated tumor-associated antigens.* Given that early clinical trial results from neoantigen-targeting vaccine have been promising, another attractive approach would be to design vaccines that can target both non-mutated TAAs and mutated cancer neoantigens [107]. Such an approach can be more effective than targeting either non-mutated TAAs or neoantigens alone. A clinical study in patients with glioblastoma recently showed that such an vaccine strategy is feasible, especially for “cold” tumors (such as glioblastoma) that contain low numbers of somatic mutations [108] and harbor fewer intratumoral tumor-reactive T cells [109]. In this clinical study [110], fifteen newly diagnosed glioblastoma patients were treated with a vaccine derived from a pre-manufactured library of non-mutated antigenic peptides (APVAC1) followed by treatment with an individualized (patient-specific) mixture of neoepitopes that were identified using a pipeline that involved analyses of the mutations, transcriptomes and immunopeptidomes of the individual patient tumors. The vaccine was administered along with poly-ICLC (polyriboinosinic-polyribocytidylic acid-poly-L-lysine carboxymethylcellulose) and GM-CSF as adjuvants. In this study robust vaccine-specific T cell responses were obtained following multiple doses of the vaccine. Generation of such a combined peptide-based vaccination strategy (readily available ‘off-the shelf’ TAA-peptides + personalized neoantigens-peptides) may be very expensive and time consuming since it involves a complex manufacturing process but might have a higher clinical success rate especially for treatment of tumors that are otherwise difficult to treat with immunotherapy.

Although these clinical results are very promising, design and manufacture of personalized vaccines can be challenging. The technical expertise and infrastructure required for generation (tumor tissue acquisition, NGS and bioinformatic pipeline) and delivery process of a personalized vaccine is enormous; the entire manufacturing process is time-consuming and requires continuous optimization which could be disadvantageous when comes to treating late-stage cancer patients with metastatic disease.

**Figure 1:**
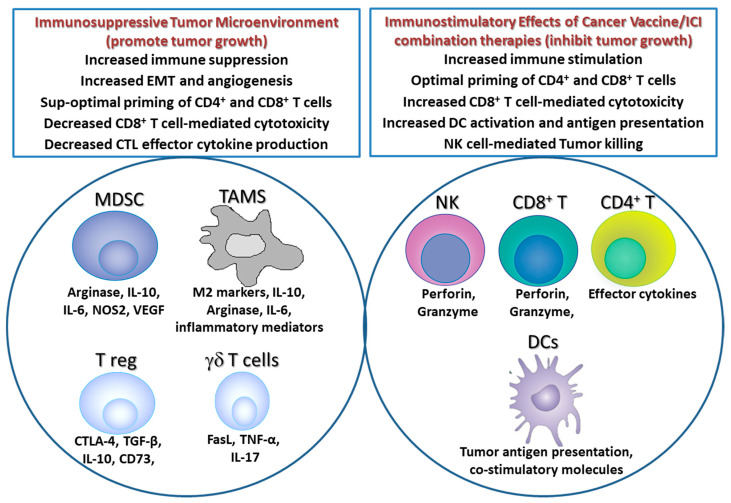
Multiple immune-inhibitory pathways operate within the tumor microenvironment that limit the efficacy of ICIs as well as cancer vaccines. A combinatorial approach consisting of cancer vaccine and ICIs can lead to optimal priming of anti-tumoral immune responses that ultimately orchestrate tumor eradication. The efficacy of this combination approach is somewhat limited by the presence of immunosuppressive cells that accumulate in the tumor microenvironment.

*(C) Combining Cancer Vaccines with Immune Checkpoint Inhibitors.* Novel immunotherapies called immune check point inhibitors, including ipilimumab (anti-CTLA-4 antibody), pembrolizumab (anti-PD-1 antibody; MERCK), and Nivolumab (anti-PD-1 antibody; BMS), have emerged as powerful therapeutic strategies for multiple types of cancer. These drugs release the brakes of the immune system and enable activation of immunity against cancer leading to improvements in survival. Recently, multiple clinical trials have tested the efficacy of combining cancer vaccines with immune checkpoint inhibitors (ICIs); such a combinatorial approach can help in overcoming the immune tolerant microenvironment prevalent in many solid tumors (Figure 1) and prime the anti-cancer immunogenicity of therapeutic vaccines. On the other hand, anti-PD-1 therapy of tumors infiltrated with sub-optimally primed T cells can lead to exhaustion of these T cells; optimal priming of these T cells by cancer vaccine treatment prior to anti-PD-1 therapy can overcome anti-PD-1-mediated T cell exhaustion and lead to tumor eradication [111] (Figure 1). This study also highlights the importance of the order of administration of ICI and cancer vaccine combination therapies [111]. The following section outlines the promising clinical studies that have tested the cancer vaccine and ICI combination therapies.

*Whole cell vaccines in combination with ICIs.* As mentioned earlier in this review, vaccines that are comprised of irradiated whole tumor cells as a monotherapy proved not to be very effective. GVAX has also been evaluated in a limited clinical setting in combination with the immune checkpoint inhibitor ipilimumab, and the results suggest that this combination therapy may be clinically beneficial for pancreatic cancer patients [112]. Currently there are ongoing phase 1 and 2 clinical trials of GVAX in various combinations with other immunotherapeutic agents such as nivolumab, urelumab and pembrolizumab [35,36]. However, the outcomes do not look promising as a Phase 2 trial using GVAX in combination with ipilumimab as a maintenance treatment for metastatic pancreatic cancer showed no improvement in overall survival compared with continuing chemotherapy treatment and in fact resulted in poorer survival than chemotherapy alone, despite promoting the differentiation of effector T cells into the memory phenotype and increasing M1 macrophages in the tumor [113]. The study was halted prematurely for futility. In a phase I trial of sipuleucel-T in combination with ipilimumab in 9 patients with docetaxel-naïve progressive mCRPC, the median survival had surpassed 50.5 months in at least 6 patients when compared with 35 months observed in phase III trials of enzalutamide or abiraterone [114]. Tumor-specific antibodies against PAP and PA2024 antigens were significantly increased in patients that received the sipuleucel-T vaccination and ICI combination therapy. These data are very encouraging and provide rationale for further validation in clinical trials with larger patient numbers.

A phase II study in previously treated melanoma patients investigated the clinical benefits of combining ipilimumab with a whole-cell DC vaccine consisting of autologous DCs electroporated with synthetic mRNA (TriMixDC-MEL) [115]. Melanoma patients were administered the combination therapy for 3 weeks followed by nivolumab treatment for 3 months (anti-PD-1 antibody). With this combination regimen, objective response rates of 38% was observed which is much higher than that obtained with ipilimumab monotherapy alone [115].

*Peptide vaccines in combination with ICIs.* Peptide vaccines have been tested in combination with ICIs including ipilimumab and nivolumab. Such a combinatorial strategy consisting of a gp100 peptide vaccine and ipilimumab was assessed as treatment strategy for patients with progressive stage IV melanoma. Patients received two different doses of ipilimumab concomitantly with the gp100 peptide vaccine. This clinical response data indicated durable objective responses in patients that was accompanied with development of autoimmunity and tumor regression [116]. This encouraging data was not reproducible in a phase III trial in metastatic melanoma patients treated with a combination of ipilimumab and gp100 peptide emulsified in incomplete Freund’s adjuvant (IFA) [117]. Patients in this trial received either ipilimumab combined with gp100 vaccine or vaccine alone or ipilimumab alone. Unfortunately, no difference in median OS was detected between the combination therapy and ipilimumab alone. One other study combined ipilimumab with a multipeptide vaccine (MART-1/gp100/tyrosinase) in the presence of an adjuvant (Montanide ISA 51 VG). In a phase I trial, patients received three different doses of the ICI inhibitor along with the multipeptide vaccine and the clinical response data from this trial showed the disease relapse rate was lower in patients that received this combination regimen that correlated with induction of autoimmunity in the treated melanoma patients [118]. This study was followed by a phase II trial wherein patients received extended-dose of ipilimumab antibody (3 or 10 mg/kg) along with the multipeptide vaccines; the clinical responses obtained with this combination are minimal and failed to improve the clinical outcomes in enrolled patients [119]. Combinations of peptide vaccines with anti-PD-1 antibody therapy has been tested in the clinical studies. Unresectable stage III and IV melanoma patients who either progressed on ipilimumab therapy or were treatment naïve were treated with a combination of nivolumab and a multipeptide vaccine (MART-1/NY-ESO-1/gp100) in the presence of an adjuvant, Montanide ISA 51 VG [120]. Nivolumab induced durable responses for up to 140 weeks in both ipilimumab-resistant and treatment-naïve melanoma patients enrolled in this study. However, no immunological responses were obtained with this combination strategy [120]. In a similar study, melanoma patients with resected IIIC to IV stages of disease were treated in an adjuvant setting with an extended dose of nivolumab (1, 3, or 10 mg/kg) in combination with a multipeptide vaccine, followed by nivolumab alone every 3 months for up to 2 years [121]. Clinical response data from this study suggested that a combination strategy with nivolumab and multipeptide vaccine can be beneficial in an adjuvant setting in melanoma and can induce both immunological and clinical responses.

Another TAA-based vaccine for prostate cancer is called PROSTVAC which is an active immunotherapy vaccine that contains prostate-specific antigen (PSA) as the tumor-associated antigen. This vaccine has been shown to generate robust T cells responses against prostate cancer. PROSTVAC is composed of two different live poxviral-based vectors: PROSTVAC-V, a recombinant vaccinia virus (rilimogene galvacirepvec), and PROSTVAC-F, a recombinant fowlpox virus (rilimogene glafolivec). These vectors harbor transgenes that code for human PSA along with three costimulatory molecules for T cells: B7.1, leukocyte function-associated antigen-3, and intercellular adhesion molecule-1, to enhance immune activation [122]. PROSTVAC has been evaluated in clinical trials in combination with GM-CSF [123,124,125]. A phase I trial assessed a combination of increasing doses of ipilimumab with PROSTVAC for patients with mCRPC. Median Overall Survival (OS) in all dose cohorts was 31.3 months which is longer that OS obtained with PROSTVAC or ipilimumab monotherapies [126]. These findings are significant, since ICI treatment alone does not show clinical benefit for patients with metastatic prostate cancer.

*Personalized Vaccines in combination with ICIs.* Most immunotherapeutic approaches to malignancies have involved promotion of immune responses against individual non-mutated tumor-associated antigens; these have generally been ineffective. Non-mutated TAA vaccines must overcome acquired tolerance of the growing tumors which can hinder the vaccine’s ability to induce robust T cell responses. However, very recently, Sahin et al. [127] reported a phase 1 clinical trial (NCT02410733) treating melanoma patients with a liposomal RNA-based vaccine (RNA-LPX; BNT111). This RNA vaccine targets non-mutated TAAs that are prevalent in melanoma (NY-ESO-1, MAGE-A3, tyrosinase and TPTE). The trial involved 89 patients with advanced melanoma who were previously treated with immune checkpoint therapy (ICI; anti-PD-1 antibody) and express at least one of the four targeted TAA. Patients received via intravenous route either BNT111 vaccine alone or a combination of vaccine and anti-PD-1 therapy. Interim data from the trial show that the vaccine induces robust CD4^+^ and CD8^+^ T cell-mediated anti-tumor immunity with durable objective responses in the vaccinated ICI-experienced melanoma patients. These results of the RNA-LPX vaccine targeting non-mutated TAAs are encouraging, and such a vaccination strategy could possibly be applied to other cancer types that express known TAAs. This study also highlights the potential of combining cancer vaccines with ICIs. A recent study by Verma et al. [111] demonstrated that PD-1 blockade of sub-optimally primed CD8^+^ T cells in cancer-bearing hosts results in dysfunctional T cells that are unable to respond to subsequent antigen/vaccine stimulation. Based on this report, it appears that combining TAA- targeting cancer vaccines with ICI therapies could be challenging and needs to be investigated further.

*(D) Vaccination against cancer with embryonic material.* Prophylactic and therapeutic cancer vaccines, as discussed above, represent an evolving type of immunotherapy that can be used to prime/boost anti-tumoral immune responses. Initial vaccine strategies involved using whole cell vaccines including irradiated, autologous tumor cells (discussed above). Potential limitations of this approach include the difficulty associated with obtaining large scale patient-specific cells and reproducibly generating vaccine preparations free of contaminants. There is abundant evidence that tumor cells and embryonic stem (ES) cells share antigens. This is because many embryonic gene products are not expressed in adult organisms and are not included in the repertoire of ‘self’ which is set by thymic selection near the end of gestation. Such ‘non-self’ gene products are immunogenic and recognized as foreign antigens. Exploiting this antigenic similarity, we and others have shown that an ES cell-based vaccine can stimulate the immune system to recognize shared oncofetal antigens and confer protection against tumors [128,129,130]. An earlier theory of oncogenesis was that cancer might arise from nests of embryonal cells, present in normal tissues and stimulated to grow by some kind of irritation. Interestingly, this theory was predictive of the discovery of cancer-initiating cells (CICs) that came over 100 years later. In an example of history repeating itself, in the mid-1960s, tumor cells and ES cells were shown to possess common gene products such as the carcinoembryonic and oncofetal antigens. During the ensuing decade, a large number of studies confirmed these findings and revealed that embryonic antigens are re-expressed in cells from solid tumors from a number of different tissues. Below, we summarize some of these very early studies with respect to cross reactive embryonic or fetal/tumor antigenicity and initial attempts to vaccinate against cancers using embryonic material-associated cancer immunity.

In the beginning of the 20th century, it was reported that prior injection of mice with fetal tissues led to rejection of transplantable tumors (reviewed in [131]). Immunization of rabbits with extracts of human gastrointestinal tumors produced antibodies which, after immunoabsorption against normal adult gut, cross reacted with GI adenocarcinomas and fetal/embryonic gut and pancreas (first reported as the so-called ‘carcinoembryonic antigens’) [132,133,134]. Interestingly, >80% of sera from humans with GI tumors and sera from women in the first two trimesters of pregnancy were found to have similar cross-reacting antibodies [133]. Subsequent investigations indicated that the presence of these “onco-fetal” antigens [135] might be almost universal. Antisera raised in rabbits against an emulsified human fetal tissue—adsorbed against adult human tissues—recognized a variety of human tumor types including skin, bronchial, renal, colonic, hepatic, lung and breast [136]. Similar to earlier observations in mice [137], no cross-reactivity with normal adult tissues except skin was found. In subsequent studies, oncofetal antigens were found in various animal and human tumor types [136,137,138,139,140,141,142,143]. A number of investigators reported that immunization of animals with early embryonic material consisting of irradiated cells from syngeneic donors would suppress or prevent the growth of transplantable tumors as well as tumorigenesis caused by viral and chemical agents [135,144,145,146,147,148,149,150]. Furthermore, immunization of hamsters with human fetal cell homogenates would prevent later SV40-mediated tumorigenesis whereas similar preparations of adult human kidney were ineffective [151]. Rats either vaccinated with embryonic material or made pregnant showed substantial suppression of pulmonary metastases [150]. These results implied that certain embryonic antigens may be sufficiently conserved between species to generate cross-reacting antibodies. It now appears that most, if not all, types of neoplastic cells express certain embryonal antigens, [131,137,138,139,141,152] a phenomenon earlier termed “retrogenetic expression” [137].

These observations support the concept that animals and humans immunized against embryonic material might be capable of recognizing and destroying neoplastic cells. Lending further strength to this concept are studies from our group which indicate that vaccination using whole ES cells, especially along with GM-CSF as an immunostimulatory adjuvant [25,153] can be very effective against lung tumors without any detectable toxicity or signs of autoimmunity [130]. The unique feature of such a strategy would be that a single whole cell vaccination system can target several fundamental cell types that are involved in the pathogenesis of cancer. Work from two other research groups [128,129] demonstrated that prophylactic vaccination of mice with irradiated xenogeneic or syngeneic ES cells is effective in preventing outgrowth of tumors in mouse models of cancer. In these studies, the precise mechanism governing ES cell-induced anti-tumor immunity as well as the identity of the target tumor antigens is still unknown; it is very likely that such an ES-based vaccination strategy will elicit T cell responses that are capable of cross-reacting with multiple tumor-expressed antigens.

Although the use of irradiated ES cell-based vaccines holds great promise for inducing anti-cancer immune responses, it also poses risk for the formation of teratomas and raises other regulatory concerns. To overcome these limitations, we asked the question: if ES cell-derived exosomes (ES-exo) can be used to exploit an already established mechanism to shuttle ES cell content intercellularly in a cell free system for inducing anti-tumor immune responses? Because a whole ES cell-based vaccine elicits potent anti-tumor immunity, we considered the pros and cons of ES-cell versus ES cell-derived exosomes therapy. Our recent work demonstrates that a cell-free vaccination strategy with exosomes derived from ES cells (ES-exo vaccine) induces potent anti-tumor effects in mice [154] and, therefore, can be considered as an attractive substitute for our whole ES cell-based vaccination approach. In contrast to whole-cell therapy, exosomes can be delivered more readily as the formulation and their stability is similar to other biologics. ES-exo are stable vesicles harboring protein and lipid contents that can be tailor-manufactured from human cell lines in clinical grade (cGMP) quality [155,156]. Furthermore, ES-exo can be produced in large quantities and cryo-preserved for more than 6 months at −80 °C with both phenotype and function intact. Our current research focus is to assess the biological features responsible for the anti-tumor activities of ES-exo vaccine as well as their immunostimulatory properties in mouse models of cancer.

*(E) Vaccination against cancer with stem cells.* Exploiting the aforementioned embryonic antigen immunogenicity, Kooreman et al. have recently showed that an induced pluripotent stem cells (iPSCs)-based vaccine can stimulate the immune system to recognize shared oncofetal antigens and confer protection against tumors [157] revealing important antigenic overlap between iPSCs and cancer cells. In their study [157], Kooreman and colleagues used a vaccination strategy that consisted of syngeneic irradiated iPSCs derived from autologous tissues in conjunction with a Toll-like receptor 9 agonist—CpG oligodeoxynucleotide (an adjuvant that elicits type I interferon responses) [157]. Immunization of mice with the iPSC vaccine either in a prophylactic or in an adjuvant tumor setting induced robust anti-tumor T cell- and B cell-mediated immune responses against both transplantable and orthotopic mouse cancer models of breast cancer, melanoma and mesothelioma [157].

Studies from our lab as well as from several other groups support the concept of antigenic similarity between stem cells and cancer cells. Leukemia stem cells in acute myeloid leukemia maintain their stemness using a transcriptional program that is also expressed in ES cells [152]. These results were in line with findings by Weinberg’s group that poorly differentiated, highly aggressive human breast cancers contain subpopulations of cells that have ES gene expression signatures. Generally speaking, this ES signature was most prevalent in estrogen receptor negative cancers in patients with very poor prognosis [158]. A popular hypothesis of tumorigenesis suggests that mutations in undifferentiated progenitor cells give rise to malignant cells that are capable of both self-renewal and differentiation. An alternative hypothesis states that cancer cells undergo progressive de-differentiation resulting in the presence of a subpopulation of cells that are capable of both self-renewal and differentiation. Regardless of the mechanism, it is now generally accepted that at least some types of cancers arise as a direct result of the self-renewal and differentiation capacities of cancer-initiating stem cells (CICs). While great progress has been made in identifying, purifying and characterizing CICs from both solid and circulating human tumor types, therapies aimed at eradicating these cells have been much slower in coming. One of the difficulties in targeting CICs is the fact that these cells make up only a very small percentage of the total cells within a neoplastic lesion. One of the first studies demonstrating the presence of cancer-initiating stem cells (CICs) in solid tumors revealed that CD44^+/^CD24^-^/ESA^+^ primary breast cancer cells initiate tumorigenesis in nude mice using as few as 200 cells while CD44^-^/CD24^+^ cells cannot, even when using up to 1000 fold greater cell numbers [159]. Since this initial report, CICs have been identified for a large number of clinically important human malignancies, including prostate, pancreatic and brain [160,161,162]. Of particular relevance is the fact that several studies now reveal that ES cell specific markers are also expressed in CICs. For example, Oct-4, Nanog, Sca-1 and Bmi-1 are all considered to be embryonic stem/progenitor cell specific markers and a number of recent studies have reported the enhanced expression of these in cancer-initiating stem cell populations [162,163,164,165,166,167]. These findings lend support to the theory that ES/iPSC cell-based vaccination induces anti-tumor immunity by eliciting anti-CIC immune responses and thus, can be a very effective strategy for targeting CICs in tumors.

## 3. Adjuvants

Many cancer vaccines are poorly immunogenic and lack clinical efficacy. To trigger more durable immune responses, adjuvants are commonly incorporated into cancer vaccine formulations to increase the efficacy of the vaccine in numerous ways, including stabilization of the antigen in the circulation, increasing the association between the antigen and APCs, and promote cytokine production which all culminate in activation of anti-cancer immune cells [168,169]. Adjuvants can be broadly characterized into 2 categories; (i) ‘Depot’ adjuvants, which are those that prolong antigen availability (e.g., mineral salts such as aluminum hydroxide, emulsions and liposomes). As part of the cancer vaccine delivery system, these adjuvants participate to enhance delivery of TAAs to APCs [170]. Examples of depot adjuvants are Montanide-based adjuvants which are water-in-oil emulsions comprised of a mineral or a metabolizable oil and a surfactant from the mannide monoleate family [171], such as Montanide ISA 51 VG, which is being tested in multiple clinical trials to boost immune efficacies of peptide-based cancer vaccines [84,120]. Other examples of depot adjuvants are mineral salts such as alum, which is however rarely used for cancer vaccines as it only triggers a Th2-mediated immune response and not a Th1 type response, which is more beneficial to eradicate tumors [172]. (ii) Immunostimulatory adjuvants including Toll Like receptor agonists, saponins, STINGS and cytokines which function as potentiators of innate and adaptive immune responses (reviewed in detail in [168]). The most extensively characterized and widely used immunostimulatory adjuvant is the cytokine GM-CSF which has been incorporated into multiple cancer vaccines including the Provenge and GVAX [173,174]. Various combinations of adjuvants and cancer vaccine strategies have been discussed in context throughout this review.

## 4. Route of Administration

Vaccines can be delivered via several routes, including intradermal, subcutaneous, intravenous and intratumoral. Intradermal injection of antigens with or without molecular adjuvants results in their rapid dispersal into the circulation due to their small size and thus they exhibit very poor specific targeting and weak accumulation in draining lymph nodes, resulting in very modest immune responses [175,176]. However, intradermal vaccination strategies have been tested in several cancers and have shown some clinical benefit [177,178,179,180,181]. Intradermal injections of a low dose of synthetic long peptides (SLPs) is safe and stimulates antigen-specific T cell responses [182] and work by targeting the vaccine to a network of cutaneous DCs which express a range of pattern recognition receptors (PRRs). Ligation of these receptors results in DC maturation and efficient priming of CD4^+^ and CD8^+^ T cells [183]. Similarly, subcutaneously delivered SLP vaccines have also achieved some measure of success in promoting a strong T cell response and mobilization of DCs [104,181,184,185]. The therapeutic efficacy of these subcutaneously administered vaccines is typically low due to their rapid diffusion into the bloodstream and subsequent systemic dispersal. This can be partly overcome by the use of depot-based adjuvants such as Freund’s adjuvant and Montanide [84], which are routinely utilized in the clinic for administration of cancer vaccines. However, subcutaneously delivered peptide vaccines often have to be delivered in milligram quantities requiring large doses of adjuvant, resulting in severe side effects [186]. Intradermal delivery of peptide vaccines uses lower doses of SLPs and thus may serve as an alternative approach to subcutaneous injection [182].

Perhaps a more unconventional approach could be to administer the vaccine intravenously. In preclinical studies, intravenous immunization of a peptide vaccine with poly-ICLC as an adjuvant generated significantly higher cytotoxic T lymphocyte (CTL) responses compared to subcutaneous delivery and resulted in more robust anti-tumor effects [187]. In addition, this study found that the use of amphiphilic antigen constructs such as palmitoylated peptides are more immunogenic that SLPs, which are routinely used in the clinic [187]. This is likely due to their ability to self-assemble into nanoparticles [188]. This strategy is still in the early stages of development and its efficacy needs to be evaluated in clinical trials.

A promising new strategy is to directly inject the vaccine into the tumor. A preliminary study in lymphoma patients using this approach used direct intra-tumoral injection of Fmslike tyrosine kinase 3 ligand (Flt3L) to recruit intratumoral DCs, local radiotherapy (XRT) to load DCs with TAA, and a TLR3 agonist (poly-ICLC) to activate DCs. This led to the accumulation of intra-tumoral cross-presenting DCs and generated a systemic anti-tumor T cell response, which eradicated all tumor cells in the body [189]. Addition of anti-PD-1 therapy in combination with this in situ approach, substantially increased the durable remission rates [189]. Although this treatment strategy is quite involved and complex, requiring multiple daily injections, and the number of study participants was small (11 patients) and not all of them responded favorably to the treatment, the results of this clinical trial (NCT01976585) were promising enough to warrant expansion of the trial to breast and head and neck cancer patients. This approach is not entirely novel. A similar strategy has been FDA approved to treat melanoma patients (T-vec or Talimogene laherparepvec). This utilizes an oncolytic virus comprised of an attenuated herpes simplex virus type 1 (HSV-1) that induces cancer cell lysis when injected in situ. Cancer cell lysis results in release of tumor antigens, virus and GM-CSF, attracting DCs, culminating in a systemic anti-tumor immune response [190,191]. Clinical trials using T-vec are ongoing but initial results are promising.

## 5. Delivery Strategies

While a large number of strategies have been developed for the preparation and formulation of cancer vaccines (whole cell-, peptide- or nucleotide-based), it still remains challenging to develop an effective common delivery platform for these vaccines that would stimulate potent anti-tumor immune responses. To date, several vaccine delivery strategies have been developed that are currently in preclinical or clinical studies, including bacterial and viral vector-based strategies, DC-based delivery strategies and biomaterial-based delivery systems, each with their pros and cons.

*Bacterial and Viral Vector-based Vaccine Strategies.* Other types of cellular vaccines include microorganisms such as bacteria and viruses which can either stimulate an immune response or deliver tumor antigens. Multiple species of bacteria have been utilized as vectors for cancer vaccines, including Bacillus Calmette-Guérin (BCG), a live attenuated strain of *Mycobacterium bovis*, *Lactococcus*, *Listeria*, and *Salmonella* [192,193,194,195,196]. For example, in preclinical models, *Listeria monocytogenes* can deliver TAAs to multiple cell types, including APCs, and induce potent antitumor immunity [197,198], however their use in a clinical setting remains inconclusive. A heterologous vaccine comprised of GVAX and *Listeria* expressing mesothelin (CRS-207) showed promise in a phase II clinical trial with advanced pancreatic cancer patients [199] but this encouraging data was not reproduced in a larger phase IIb study [200], resulting in development of CRS-207 being abandoned (https://investors.aduro.com/news-releases/news-release-details/aduro-biotech-provides-update-crs-207-programs, accessed on 21 May 2021). The use of Listeria monocytogenes as a vector for cancer vaccines is reviewed more comprehensively elsewhere [196].

Several viruses have also been utilized as cancer vaccine vectors [201]. The most commonly used viral vaccine vectors are derived from poxviruses, adenoviruses and alphaviruses [201]. The advantage of using virus-based vaccines is the immune system efficiently responds to viruses by eliciting both durable adaptive and innate responses. A disadvantage of viral vectors, however, is that the immune response neutralizes the vector, limiting repeat vaccination. This can be overcome by using a heterologous prime-boost strategy where a TAA is initially delivered by one viral vector, followed by a subsequent boost with the same TAA using a different viral vector or vector type (such as a plasmid). An example of this is PROSTVAC-VF, which uses PSA encoded by a vaccinia virus to prime, followed by six booster doses with PSA delivered by a fowlpox virus [202]. Although PROSTVAC showed an increase in overall survival in phase II clinical trials in men with castration resistant prostate cancer (CRPC) [203], this was not reproduced in a larger phase III study [125] and consequently the trial was prematurely ended (2010-021196-85). A further example of this strategy is ALVAC, a cancer vaccine incorporating various TAAs in a canarypox vector, which has been studied in melanoma, colon cancer and ovarian cancer patients [204,205,206,207]. However, these studies utilized relatively small patient numbers resulting in several being terminated (ClinicalTrials.gov accessed on 24 May 2021). More recently, a vaccine strategy has been developed using a chimpanzee adenovirus encoding 3 prostate cancer antigens (PSA, PSMA and PSCA) for priming, followed by boosts with DNA, encoding the same 3 TAAs together with low dose anti-CLA4 antigen administered proximal to the vaccine [208]. In preclinical models, this strategy resulted in potent anti-tumor antigen T- and B-cell responses and administration of the checkpoint inhibitor locally appeared to be more effective than systemic delivery at enhancing the vaccine-induced T-cell responses. This therapeutic regimen has recently entered clinical trials. The use of virus vector-based vaccine strategies has been extensively reviewed in other publications [201,209,210].

*Ex Vivo-Pulsed DC*. Due to the critical role DCs play in antigen presentation, they have been exploited for cancer immunotherapy. In this approach, DCs are loaded with tumor antigens ex vivo and then injected into cancer patients to induce anti-tumor T cells responses [211]. Several clinical trials have been conducted on DC vaccines [106,212,213,214,215] and while results from these studies are encouraging, only a small percentage of patients achieved robust objective clinical responses [216]. This may, in part, be due to the use of monocyte-derived DCs (MoDCs), which have been reported to have decreased migratory capacity towards the site of T cell interactions, probably caused by their extensive ex vivo manipulation [216,217]. Naturally circulating DCs (nDCs) may be a much more effective alternative to MoDCs, however they only constitute about 1% of blood mononuclear cells making their use extremely challenging [216,218]. The use of ex vivo-pulsed DC vaccines is costly, labor intensive and requires specialized manufacturing, thus limiting their broad clinical applicability [219] and more extensive studies are warranted on this therapeutic approach.

*Nanoparticles.* Nanotechnology has been extensively studied in biomedical research and drug delivery. Nanostructures typically have a diameter of less than 1µM and are 100–10,000 times smaller than a mammalian cell [220,221,222]. Nanosystems have adaptable material and surface functionalization properties, making them extremely versatile drug delivery systems. Several nanosystems have been evaluated as a vaccine delivery mechanism for cancer vaccines, including polymeric nanoparticles, liposomes and magnetic nanoparticles [220]. The most widely used polymers for delivering therapeutic biomolecules are Poly(d,l-lactic-co-glycolic) acid (PLGA) and poly(lactic acid) (PLA) [220]. Preclinical studies have shown that poly(lactic-co-glycolic acid) (PLGA) capturing nanoparticles can efficiently deliver TAAs to APCs resulting in expansion of cytotoxic CD8^+^ T cells and improved response to immunotherapy [223]. Currently, there are very few clinical studies evaluating nanoparticle delivered cancer vaccines. An ongoing phase I clinical trial (NCT03313778) in patients with solid tumors, including melanoma, NSCLC, breast, prostate, and cervical, is testing the safety, tolerability and immunogenicity of Lip, a lipid encapsulated personalized cancer vaccine. Preliminary data suggest this regimen is safe and well tolerated and clinical responses have been observed in combination with pembrolizumab [224]. To date, only one nanoparticle cancer vaccine has reached phase III clinical trial (NCT00409188). Tecemotide (L-BLP25), is a MUC1 antigen liposome-based vaccine [225], however, no difference in overall survival was found between the vaccine and placebo in patients with stage III NSCLC [226]. Similar disappointing clinical outcomes were observed in a phase II trial in breast cancer patients [227]. While nanocarriers show a favorable safety profile, are FDA-approved, and provide a versatile tool for biomolecule delivery, their use as delivery agents for cancer vaccines still requires further investigation.

Recent advances in nanotechnology has led to the use of nanomaterials to improve DC activation and DC-mediated tumor-specific T cell responses [228]. In preclinical mouse models of cancer, RNA-lipoplexes (RNA-LPX) encoding viral antigens or neoantigens triggers T cell effector responses, IFN-α release from DCs and macrophages resulting in DC maturation and activation, and subsequent IFN-α-mediated tumor rejection. In a phase I dose escalation study of melanoma patients, a low dose of these RNA-LPXs induced IFN-α and strong antigen-specific T cell responses [229].

Although nanosystems offer versatile vaccine delivery approaches, they can be limited by the complexity of material production which may affect stability of the antigen and immunogenicity. An alternative simpler approach to deliver TAAs and neoantigens peptides is to use plasma membrane vesicles (PMVs) derived from biological materials such as cultured cells, which can be modified to include specific TAAs together with co-stimulatory molecules [230]. This approach has been successfully utilized in preclinical studies wherein PMVs were loaded with a glycosylphosphatidylinositol (GPI)-anchored form of the breast cancer antigen HER-2. Mice immunized with these modified PMVs showed strong HER-2-specific antibody responses that translated to breast cancer protection in these mice. Incorporation of immunostimulatory molecules such as IL-12 and B7.1 into these PMVs enhanced anti-tumor T cell mediated effector responses [230].

Select clinical trials discussed in this review are summarized in Table 2.

## 6. Conclusions

While immunotherapy has offered cancer patients new hope, it is unfortunately not effective for all patients. Despite the immense potential of cancer vaccines for tumor immunotherapeutics and the overwhelming amount of evidence that cancer vaccines are able to activate the immune system and generate antitumor activity in some patients, a host of reasons likely contribute to their inability to achieve their full potential as standalone cancer therapies, including choice of tumor antigen, immune tolerance mechanisms and the development of an immunosuppressive tumor microenvironment. However, cancer vaccines offer an attractive approach to synergize with currently available immunotherapeutics strategies, such as immune checkpoint inhibitors, oncolytic viruses and other immune modulators such as several cytokines, to boost anti-tumor immunity. Such a combinatorial therapeutic regimen has the potential to compensate for the shortcomings of each therapy when used individually. This approach will likely offer clinical benefits to patients with advanced metastatic disease. Prophylactic vaccines that can elicit preemptive immunity could significantly reduce the risk of oncogenesis by overcoming the major problems associated with successfully treating advanced and metastatic disease. Large-scale clinical trials to test these prophylactic strategies would first need to be conducted, which are costly and challenging. Nevertheless, both prophylactic and therapeutic cancer vaccines have been successfully developed and approved. Recent clinical trial data using personalized cancer vaccines are highly encouraging, and perhaps we should keep the balloons on hand.

## Figures and Tables

**Table 1:** Various anti-cancer vaccines including prophylactic and therapeutic vaccination strategies.

Cancer Vaccine	Strategy	Vaccine Type	Advantages	Disadvantages
Preventive	Viral antigens-based vaccines	HBV HPV (Gardasil, Cervarix)	Highly efficacious Excellent safety profile Highly immunogenic	Restricted to cancers with known etiopathogenic agents
	Retired antigens-based vaccines	AMHR2-ED α-lactalbumin	Specific for adult onset non- viral associated cancers Highly specific Immunogenic	Only applicable to cancer types with known retired antigens
	Embryonic material-based vaccines	Intact ES cells Intact IPSCs ES cell exosomes	Comprehensive immune responses against multiple antigens; Broad spectrum (off-the shelf)	Complex and costly manufacture procedure
Therapeutic	Cell-based vaccines	Gvax Sipuleucel-T Algenpantucel-L STINGVAX	High antigenic immunogenic potency; Control of antigen presentation	Risk for vaccine-triggered adversary effects; Complex and costly manufacture procedure
	Viral vector- or bacterial vector-based vaccines	PROSTVAC ALVAC	High antigenic immunogenic potency; Broad spectrum (off-the-shelf); Suitable for large-scale manufacture	Host-induced immune responses to vectors; Safety concerns for accidental infection; Risk for vaccine-triggered adversary effects
	Peptide-based vaccines	CTAG1B MAGE-A3 BIRC5 WT1 Peptide-based mutant neo-epitopes (personalized vaccines)	Low risk for vaccine-triggered adversary effects; Suitable for large-scale manufacture	Modest antigenic immunogenic potency; Restriction in HLA haplotype subtype
	DNA- or RNA-based vaccines	RNA-based neo-epitopes (personalized vaccines) RNA-based TAAs (NY-EXO-1, MAGE-A3, Tyrosinase)	Flexible to deliver multiple antigens; No restriction in HLA haplotype subtype; Comprehensive T cell and B cell responses; Suitable for large-scale manufacture	Modest antigenic immunogenic potency; Stringent temperature requirements for storage and transport of RNA-based vaccines

**Table 2:** Select clinical trials utilizing various cancer vaccine strategies.

Number	Phase	Cancer Type	Vaccine	Outcome	Reference
NCT00089856	III	Metastatic Prostate Cancer	Allogeneic prostate cancer cells overexpressing GM-CSF (GVAX)	Terminated (<30% chance of meeting primary endpoint)	[32]
NCT01836432	III	Metastatic Pancreatic Cancer	Pancreatic ductal adenocarcinoma cells expressing aGT (Algenpantucel-L)	No improvement in overall survival	[35]
NCT00676507	III	Advanced NSCLC	Allogeneic NSCLC cells with reduced TGFβ2 expression (belagenpumatucel-L)	Terminated without meeting the survival endpoint	[55]
NCT00796445	III	Melanoma	Recombinant MAGE-A3 and AS15 immunostimulant	Terminated early for the lack of efficacy	[71]
NCT00409188	III	Stage III NSCLC	Lipopeptide with MUC1 peptide sequence (Tecemotide)	Clinically relevant prolonged overall survival	[82]
NCT00683670	I	Advanced melanoma	Autologous dendritic cells loaded with patient-specific neoantigens	Diverse neoantigen-specific T cell receptor repertoire	[104]
NCT01970358	I	Advanced melanoma	Twenty predicted personal tumor neoantigens	Induction of CD4+ and CD8+ T cells targeting neoantigens	[105]
NCT02035956	I	Advanced melanoma	Poly-neoepitopic coding RNA of an individual patient	T cell responses against multiple vaccine neoepitopes	[106]
NCT02149225	I	Newly diagnosed glioblastoma	Unmutated antigen library and personalized neoepitopes	Sustained CD8+ and CD4+ T cell responses	110
NCT01896869	II	Metastatic Pancreatic Cancer	GM-CSF-secreting allogeneic pancreatic tumor cells and ipilimumab	No improvement in overall survival	[113]
NCT01832870	I	metastatic prostate cancer	Autologous dendritic cells loaded with PA2024 (Sipuleucel-T) and ipilimumab	Increase in tumor-specific antibodies	[114]
NCT01322490	III	Metastatic prostate cancer	Poxviruses expressing PSA and costimulatory molecules (PROSTVAC)	No effect on alive without events and overall survival	[118]
NCT00113984	I	Metastatic prostate cancer	PROSTVAC and ipilimumab	Enhancement in co-stimulation of the immune system	[119]
NCT01302496	II	Advanced melanoma	Autologous dendritic cells electroporated with synthetic mRNA and ipilimumab	Highly durable tumor responses	[120]
NCT00094653	III	Metastatic melanoma	Glycoprotein 100 peptide and ipilimumab	No effect in overall survival	[122]
NCT00084656	II	Advanced melanoma	Multipeptides (tyrosinase/gp100/MART-1) and ipilimumab	No improvement in the clinical outcomes	[124]
NCT01176461	I	Advanced melanoma	Multipeptides (MART-1/NY-ESO-1/gp100) and Nivolumab	No immunological responses obtained	[125]
NCT02410733	I	Advanced melanoma	Liposomal RNA of 4 non-mutated TAAs (NY-ESO-1/MAGE-A3/tyrosinase/TPTE) and anti-PD-1 antibody	Strong CD4+ and CD8+ T cell immunity against antigens	[127]
NCT01976585	I	Low-Grade Lymphoma	Flt3L, radiotherapy, and TLR3 agonist	Increase in the durable remission rates	[189]
NCT03313778	I	Melanoma	Lipid-encapsulated mRNA	Safe and well-tolerated	[224]
NCT00409188	III	NSCLC	MUC1 liposomal-based vaccine	No difference in overall sutvival	[226]

## Data Availability

Not applicable.
